# A Phase II Study of ^177^Lu–Lilotomab Satetraxetan, a CD37 Antibody–Radionuclide Conjugate, as Third- or Later-Line Treatment of Rituximab-Refractory Follicular B-Cell Lymphoma Patients

**DOI:** 10.3390/ph19020250

**Published:** 2026-02-01

**Authors:** Roy H. Larsen, Arne Kolstad, Alexander Fosså, Ada Repetto-Llamazares, Knut T. Smerud, Timothy Illidge, Øyvind S. Bruland

**Affiliations:** 1 NucliThera AS, 0884 Oslo, Norway; sciencons@gmail.com (R.H.L.); oyvind.bruland@medisin.uio.no (Ø.S.B.); 2 Innlandet Hospital, 2819 Gjøvik, Norway; arne.kolstad@sykehuset-innlandet.no; 3 Norwegian Radium Hospital, Oslo University Hospital, 0379 Oslo, Norway; aff@ous-hf.no; 4 Smerud Medical Research International AS, 0274 Oslo, Norway; knut.smerud@smerud.com; 5 Christie NHS Foundation Trust, Manchester NIHR Biomedical Research Centre, University of Manchester, Manchester M20 4BX, UK; tim.illidge@manchester.ac.uk; 6 Institute of Clinical Medicine, Faculty of Medicine, University of Oslo, 0372 Oslo, Norway

**Keywords:** CD37, single-dose radioimmunotherapy, follicular lymphoma, Phase IIB

## Abstract

**Background**: CD37, an antigen highly expressed in B-cell malignancies, served as the target in the LYMRIT-37-01 Part B (PARADIGME) and Part C studies employing a single intravenous injection of the radioimmunoconjugate ^177^Lu–lilotomab satetraxetan (Betalutin^®^). **Methods**: Patients with follicular lymphoma (FL), grades I–IIIa, who had received at least two previous lines of therapy and were refractory to at least one previous regimen with rituximab or an anti-CD20 agent, were included. They were randomized to receive either a 40 mg lilotomab pretreatment and an activity dosage of 15 MBq/kg Betalutin (“40/15” regimen) or 100 mg/m^2^ of lilotomab and 20 MBq/kg of Betalutin (“100/20” regimen). In total, 109 patients were enrolled and received Betalutin, 72 of whom received the 40/15 regimen and 28 received the 100/20 regimen. An additional heavily pretreated “special population” of nine patients received 40/12.5 (i.e., a reduced Betalutin dosage) due to low platelets and/or a previous autologous stem cell transplant. Part C was a small expansion cohort of four patients, all receiving the 40/15 regimen, and was designed to obtain supplementary pharmacokinetic data. **Results:** The efficacy analysis set comprised a total of 100 patients from the PARADIGME study. The overall response rates were 38.9% and 32.1%, and the complete response rates were 20.8% and 14.3% in the 40/15 and 100/20 groups, respectively. Correspondingly, the median response durations were 8.5 months and 3.4 months in the two groups. Hence, increasing the Betalutin activity dose by using the stronger protective CD37 pre-dosing (“100/20”) did not improve the therapeutic benefit. The most common grade ≥ 3 adverse events were hematologic, including neutropenia (11.5%) and thrombocytopenia (8.0%), with nadirs occurring around weeks 5–7 and recovery by week 11. **Conclusions:** A single-dose administration of Betalutin had a mild toxicity profile with a clinically relevant response rate. It represents a viable treatment alternative in FL patients who are not suitable for more toxic and long-lasting treatments. Trial Registration: This trial was registered at clinicaltrials.gov under #NCT 01796171.

## 1. Introduction

Follicular lymphoma (FL) is the second most common subtype of non-Hodgkin lymphoma (NHL) in the Western world, with an annual incidence of approximately 2–5 cases per 100,000, and this rate is increasing [1,2,3]. FL represents about 20% of all NHL [4] and is currently considered an incurable B-cell lymphoid neoplastic disease. As opposed to grade IIIB, which is aggressive, FL grades I–IIIa are considered an indolent disease and are often asymptomatic and discovered incidentally. Even when the disease reaches an advanced stage, many patients do not require immediate systemic treatment. The median age at diagnosis is 63 years, the median overall survival is 14 years, and 90% of patients survive 5 years or more after diagnosis [2,5,6]. Although therapies are available, FL is characterized by successive relapses, becoming increasingly refractory to treatment with shorter remission periods and an increased risk of accumulated toxicity [7,8,9]. Among the elderly, in particular, this represents a significant burden and impacts quality of life, underlining the importance of tailored treatment plans [10].

Current treatment guidelines recommend chemoimmunotherapy for the frontline treatment of FL, namely, an anti-CD20 monoclonal antibody (rituximab) with bendamustine or CHOP (cyclophosphamide, doxorubicin, prednisone, vincristine) chemotherapy [11,12,13]. Such frontline therapy achieves overall response rates (ORRs) approaching 100% [14]. For patients who respond, rituximab maintenance therapy may be considered, although it has not been universally adopted due to concerns related to an increased risk of infections and the lack of a convincing benefit for overall survival. Viable chemotherapy-free alternatives for previously untreated FL patients include single-agent rituximab or rituximab and lenalidomide combined [15,16].

However, approximately 20% of FL patients progress early, within 24 months—the so-called POD24—whereas others become refractory to treatment later [17,18,19]. For POD24 patients or those with refractory FL after several lines of therapy, the prognosis is poor. Significant unmet medical needs remain, especially for individuals who experience histological transformation into a more aggressive lymphoma subtype [17]. Similarly, frail, elderly patients or others with comorbidities, who cannot tolerate intensive chemotherapy, also urgently need new treatment options. The standard of care for patients with relapsed or refractory (r/r) FL, especially after failing two lines of prior treatment, is evolving quickly with increasing use of bispecific antibodies and CAR-T cells [20,21]. When PARADIGME began, the options of rituximab or obinutuzumab in combination with chemotherapy, radioimmunotherapy (RIT), kinase inhibitors, and, for selected patients, stem cell transplantation (SCT) were available [15,22]. The treatment goals for this population remain the preservation of quality of life and the prolongation of progression-free survival (PFS) [23].

As FL is a radiosensitive disease, RIT using a radiolabeled monoclonal antibody to specifically target an antigen expressed on tumor B cells has previously been widely studied as an appealing approach [24]. RIT offers single dosing and would be particularly beneficial to patients with significant comorbidities and/or mobility restrictions. However, only specialized centers with a nuclear medicine team are equipped to deliver such therapy. At the time the current study was planned, two RIT-based treatments were available. ^90^Yttrium ibritumomab tiuxetan (Zevalin) was approved in Europe for the treatment of adults with rituximab-relapsed or -refractory CD20+ follicular B-cell NHL. However, the authorization lapsed in January 2024, after it had not been marketed for more than three consecutive years; consequently, this option is no longer available for this patient population. Similarly, 131-iodine tositumomab (Bexxar), which was available in the US for the treatment of NHL, was withdrawn from the market in 2014 due to very low sales. These RITs produced high rates of response [25], but because both RITs target the CD20 antigen, their efficacy may be reduced in FL patients experiencing subsequent disease progression following previous treatments with rituximab. A study with ^131^I-labeled anti-CD37 radioimmunoconjugate (RIC) with high dose and bone marrow support indicated relevant clinical benefits, but the study was stopped because greater benefits were derived from an anti-CD20 RIC [26]. Later in vivo data indicated that the tumor retention of radioiodine from anti-CD37 RICs was poor, probably due to internalization-driven dehalogenation [27].

Therefore, a radiometal-based anti-CD37 RIC was developed using DOTA-conjugated ^177^Lu, a beta-emitter that was deemed appropriate for the internalizing CD37 antigen [28]. To circumvent the problem of resistance to anti-CD20-based treatment, a novel RIT targeting the CD37 antigen, ^177^Lu–lilotomab satetraxetan (Betalutin^®^), entered into clinical development [29]. The CD37 target is an internalizing and highly glycosylated transmembrane protein selectively expressed by normal B cells and on the cell surface of tumors of B-cell origin [28,30,31,32]. Through its radioactive payload, Betalutin delivers a cytotoxic dose of radiation closer to the nucleus, affecting the deoxyribonucleic acid (DNA) of tumor cells. Lutetium-177 was chosen because it is a commercially available beta-emitter with a half-life of 6.7 days and a mean beta energy of 0.15 MeV and similarly emits imageable photons, thus enabling biodistribution and dosimetry measurements [33,34]. Preclinical studies showed excellent therapeutic efficacy in murine NHL models and demonstrated that targeting CD37 was superior in specifically killing tumor cells in vitro and in vivo compared with ^177^Lu-labeled rituximab targeting CD20 [28,35,36,37].

LYMRIT-37-01 was a first-in-human study in three parts (A, B, and C) with Betalutin that covered a range of early clinical-stage aspects (clinicaltrials.gov #NCT01796171). Part A was a Phase I/IIa study in relapsed/refractory indolent NHL (FL grades I–IIIA, marginal zone, small lymphocytic, lymphoplasmacytic, and mantle cell lymphoma) that defined the maximum tolerated dose of Betalutin and the optimal pretreatment and pre-dosing regimen(s) for expansion into the Phase IIa part of the study. In Part A, multiple dose levels were tested, including arms without pre-dosing and arms with lilotomab pre-dosing; the 40 mg fixed and 100 mg/m^2^ body surface area (BSA) pre-dosing regimens were selected for further study based on favorable tumor-to-red marrow dose ratios and an acceptable hematologic safety profile. Encouraging efficacy results (ORR 65%, complete response rate (CRR) 30%) were found within the FL subgroup of Part A, including a favorable hematological toxicity profile that has previously been published [29].

Hence, PARADIGME (Part B) focused solely on FL patients and was designed as a randomized Phase IIb dose-finding study to further delineate the risk/benefit profile in a population of relapsed FL patients who had received at least two prior lines of systemic treatment and were refractory to at least one previous regimen that contained rituximab or an anti-CD20 agent. A further small expansion cohort (Part C) in relapsed indolent NHL was initiated to obtain supplementary pharmacokinetic data due to the limited availability of these data from Parts A and B. The present paper reports safety and pharmacokinetic data from Parts B and C, as well as the efficacy data from Part B.

## 2. Results

### 2.1. Patient Characteristics

The study was terminated prematurely prior to completion of the planned enrollment and follow-up, as detailed in the Section 4. Consequently, the results presented here are based on data collected up to the time of termination and reflect a reduced study population and shorter observation period than originally planned. This context should be considered when interpreting the findings reported below.

**Figure 1:**
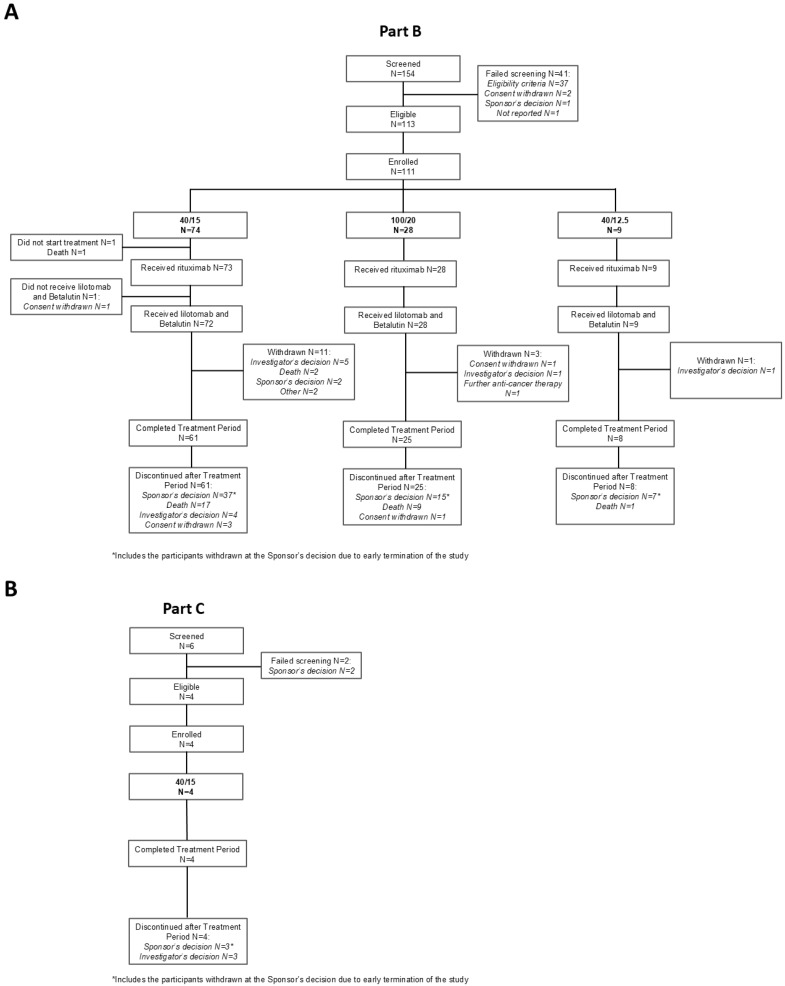
Participant disposition for LYMRIT-37-01 (**A**) Part B (PARADIGME) and (**B**) Part C.

The patients were enrolled in the study between April 2018 and June 2022. Figure 1 shows the detailed disposition of patients for Parts B and C. A total of 154 patients were screened for PARADIGME (Part B), 113 of whom were eligible for enrollment. Two eligible patients were not enrolled, one due to rapid disease progression and one due to being outside the time window for treatment because of the COVID-19 pandemic. Of the 111 enrolled patients, 1 died before receiving any study intervention, and 1 withdrew consent after receiving pretreatment with rituximab only. Thus, 109 patients received one of the three study interventions: 72 patients received the 40/15 regimen, 28 received the 100/20 regimen, and 9 received the 40/12.5 regimen. Of these, 11 patients belonged to the special populations: 5 patients with low platelets, 5 patients with a prior autologous SCT, and 1 patient with both. The majority (94/109) completed the 3-month treatment period as per the protocol, but due to the sponsor’s decision to prematurely terminate the study, 59 of those patients did not subsequently contribute to an analysis of sustained efficacy. Among those patients who were not censored due to the premature termination, 27 died whilst in study follow-up. For Part C, six patients were screened, four of whom were eligible and included and completed the treatment period of 3 months.

The key demographics and baseline characteristics of the efficacy analysis set are presented in Table 1. There were no marked differences between the safety and efficacy analysis sets. The two dosing cohorts of the efficacy set were generally well balanced in terms of demographics, baseline FL disease characteristics, and number and type of previous therapies. Overall, there were slightly more females than males enrolled, and the median age was 69 years (range 44–89) at the time of enrollment. Sixty-five percent of the patients were 65 years or older. The median time since initial diagnosis of FL was 72.7 months, with a median of 1.4 months since last relapse. The four patients in cohort C included two with FL and two with marginal zone lymphoma; they had a shorter median time since initial diagnosis (60.7 months) than the Part B population and a median of 1.9 months since last relapse.

### 2.2. CD37 Expression

All the biopsy samples were positive for CD37, with the exception of one small and fragmented sample. The predominant staining was of high or medium intensity (47 and 41 of 99 samples, respectively). The majority of samples had a homogeneous CD37 expression (73 of 99).

### 2.3. Treatment Efficacy

In the efficacy analysis set, the ORR was 38.9% and 32.1% with CRRs of 20.8% and 14.3% in participants receiving 40/15 and 100/20, respectively (Table 2). For the 55 of 100 patients who were double refractory (i.e., to both an anti-CD20-based regimen and an alkylating agent), the ORR was 29.1%, and the CRR was 14.5%, whereas for the 40/15 regimen within this double refractory subgroup, the ORR was 30.6% and the CRR was 16.7%. For the 11 patients in cohort B who composed the special population with widened inclusion criteria, the ORR was 22.2% in the nine patients dosed with the 40/12.5 regimen, whereas one patient achieved a CR after the 40/15 regimen.

**Figure 2:**
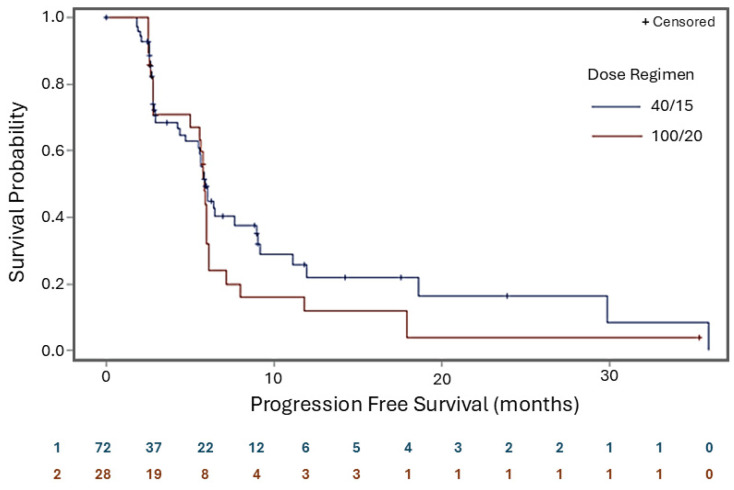
Progression-free survival LYMRIT-37-01 Part B (PARADIGME). Kaplan–Meier plot including number of patients at risk—efficacy analysis set.

As shown in Table 3, the median duration of response (DoR) was 8.0 months for the 40/15 cohort and 3.4 months for the 100/20 group. The median duration of complete response (DoCR) was 8.5 months for the 40/15 group and 9.2 months for the 100/20 group. After 15 months, 43.9% of the responders and 46.7% of the complete responders in the 40/15 treatment group were still responding. The figures for the 100/20 group were 22.2% and 25.0%, respectively. The median PFS was 5.9 months versus 5.8 months for the 40/15 and 100/20 groups, respectively. Figure 2 shows the Kaplan–Meier plot for the PFS.

### 2.4. Safety

Treatment-emergent adverse events (TEAEs) were reported in 94 (83.2%) participants from Day 1 until the end of the study, and those with an incidence of 3% or more are shown in Table 4. The most common TEAEs were cytopenias, with thrombocytopenia in 19 (16.8%) and neutropenia in 18 (15.9%) patients. Anemia and fatigue were also reported in >10% of participants. There were a total of 29 serious adverse events (SAEs) in 19 (16.8%) participants. The overall incidence of SAEs was 15.8% in the 40/15 group and 25.0% in the 100/20 group. There were two fatal SAEs in participants receiving 40/15 (pancreatic adenocarcinoma and COVID-19), neither of which was considered to be related to the study intervention. The majority of adverse events were Common Terminology Criteria for Adverse Events (CTCAE) grade 1 or 2, whereas 42.5% of patients reported grade 3–5 TEAEs, with neutropenia (11.5%) and thrombocytopenia (8.0%) being the most frequent.

Betalutin-related TEAEs were reported in 60 (53.1%) patients. There was one case of TEAE of myelodysplastic syndrome, leading to withdrawal from the study. A total of six adverse events of special interest (AESIs) were reported, two of which (i.e., endometrial cancer and myelodysplastic syndrome) were assessed to be possibly related to Betalutin.

**Figure 3:**
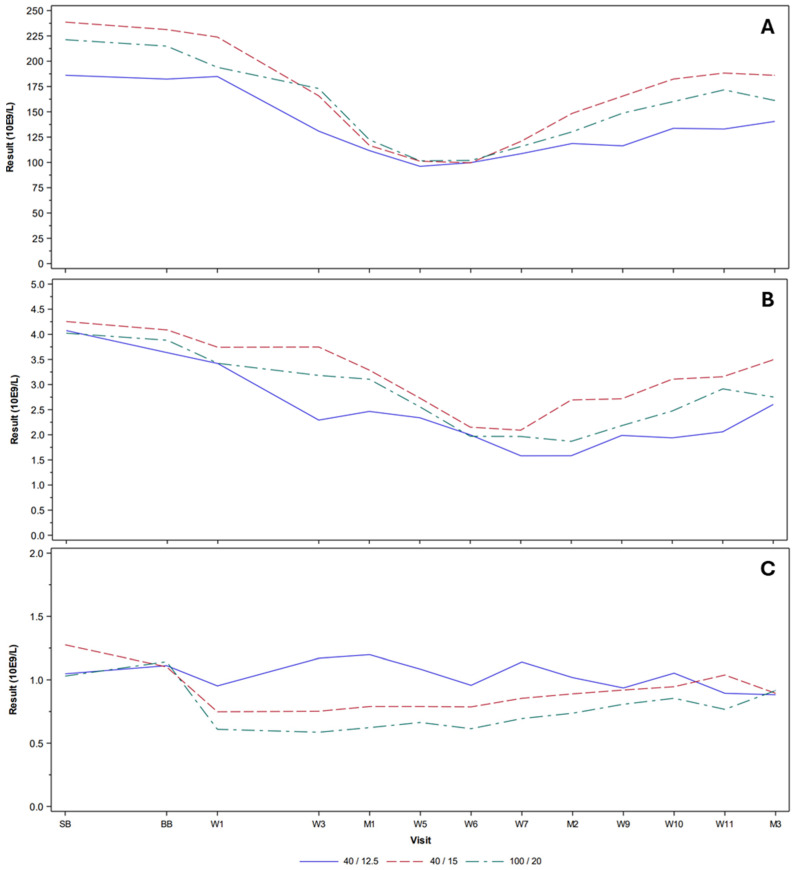
Hematological parameters over time LYMRIT 37-01 Part B and Part C—safety analysis set. (**A**) Platelets, (**B**) neutrophils, and (**C**) lymphocytes. SB = study baseline; BB = Betalutin baseline; W = week; M = month.

Overall, reductions in lymphocyte count to <0.2 × 10^9^/L were observed in seven (6.2%) patients. Reductions in neutrophil count to <1 × 10^9^/L and <0.5 × 10^9^/L were observed in 36 (31.9%) and 12 (10.6%) patients, respectively, for the 40/15 and 100/20 regimens. Reductions in platelet count to <50 × 10^9^/L and <25 × 10^9^/L were observed in 24 (21.2%) and 8 (7.1%) patients, respectively. The mean platelet, neutrophil, and lymphocyte counts over time are shown in Figure 3. A nadir appeared at around weeks 5–7, but values returned to baseline at around week 11.

### 2.5. Pharmacokinetics

**Figure 4:**
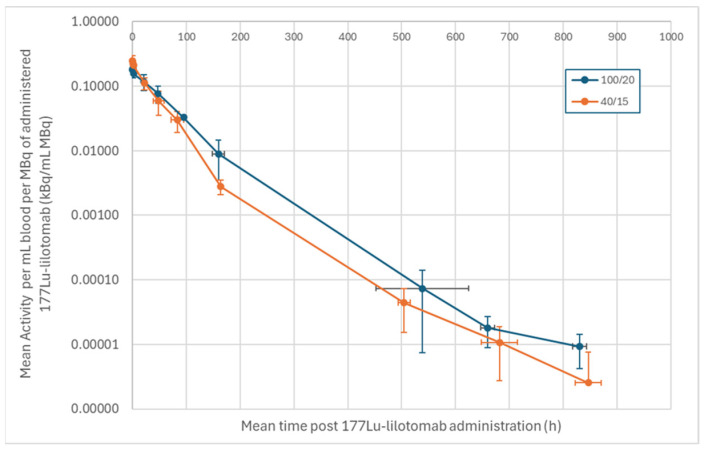
Mean radioactivity content per mL of blood per ^177^Lu–lilotomab MBq administered as a function of time after ^177^Lu–lilotomab administration for LYMRIT-37-01 Part B and Part C.

Pharmacokinetic data were available from four patients receiving the 100/20 regimen (Part B) and six patients receiving the 40/15 regimen (three from Part B and three from Part C). No significant differences were observed in the pharmacokinetic profiles of the activity doses, that is, the 100/20 and 40/15 regimens (Figure 4).

## 3. Discussion

The PARADIGME study represents the first large clinical study of anti-CD37 RIT using a radiometal isotope. The current study involves patients with FL who had received at least two prior systemic anti-neoplastic or immunotherapy-based regimens and who were refractory to at least one previous regimen that contained rituximab or another anti-CD20 agent. With the 40/15 Betalutin regimen in this Phase 2b study, an ORR of 38.9% and a CRR of 20.8% were obtained. However, the ORR was significantly lower than that found for the FL patients in the preceding Phase 2a, Part A, study [29], which had an ORR of 58% and a CRR of 19%. The DoR in PARADIGME was 8.5 months for both overall responders and complete responders, as opposed to 13.6 months in Part A. Moreover, the median PFS in PARADIGME was inferior to Part A, at 5.8 vs. 9.0 months, respectively.

This difference in efficacy between the two parts of the same umbrella study is most likely attributed to the more heavily pretreated patient population in PARADIGME. The median number of prior treatments in Part A was 2 (range 1–7), whereas in PARADIGME, it was 3 (range 1–12). Further, 98.6% of the patients in PARADIGME had received ≥2 prior regimens compared to 71.9% in Part A. The proportion of patients who were refractory to their last therapy was significantly higher in PARADIGME (72.2%) than in Part A (28.1%). Furthermore, 97.2% of the patients in PARADIGME were rituximab-refractory compared to 37.5% in Part A. The median time from the last previous treatment to study intervention was notably shorter in PARADIGME (9.9 months) than in Part A (32.8 months). These differences in patient characteristics suggest that the lower efficacy observed in PARADIGME may be due to the generally more aggressive nature of the disease, signified by the heavily pretreated patient population, which could impact the overall response to the anti-CD37 RIT. The CD37 staining results from the Part B and C studies confirmed that CD37 was well expressed in the study population and consistent with previous findings in Part A [29] and in previously published studies [28]. All the patient samples (with the exception of one small and fragmented sample) demonstrated positive staining, underscoring the strong and consistent expression of the target, CD37. Hence, the CD37 expression could not account for the reduced efficacy observed in the PARADIGME study vs. Part A.

As previously published in Part A [29], rituximab alone, given 28 and 21 days before Betalutin administration, did not result in a satisfactory tumor-to-bone marrow radiation dose ratio, while pre-dosing with lilotomab significantly improved this ratio. Based on this finding, the pre-dosing with rituximab was simplified to one treatment at −14 days to remove circulating B cells, which could potentially otherwise trap Betalutin before it reached the tumor [29]. In Part A, it was shown [29] that patients receiving pre-dosing with 40 mg of lilotomab or a pre-dosing of 100 mg/m^2^ experienced considerably better tumor-to-bone marrow radiation dose ratios than those with no pretreatment [33]; therefore, pre-dosing with lilotomab before Betalutin administration was made a part of the dosing regimen in Parts B and C. To indicate the difference in the amount of lilotomab between 40/15 and 100/20 in terms of the injected monoclonal antibody dose, 100/20 would be approximately a factor of 3 higher than 40/15. As demonstrated in Figure 2, there is a tendency toward a better long-term overall effect with the lower pre-dosing with unlabeled antibody and reduced dosage of Betalutin than with the higher pre-dosing. This tendency may relate to the blocking effects of tumor cells, especially in smaller lesions, by the higher concentration of unlabeled antibody. Thus, 40 mg lilotomab pre-dosing seems to be a reasonable compromise between bone marrow protection and good access to the CD37 antigens on the tumor cells. Like Betalutin, the previously approved RICs ^90^Y-ibritumomab tiuxetan (Zevalin) and ^131^I-tositumomab (Bexxar) utilize pre-dosing with unlabeled antibodies to improve the biodistribution and radiation dose distribution. Our findings with Betalutin in this study are indeed comparable to those reported for anti-CD20 RIT with ^90^Y-ibritumomab tiuxetan in rituximab-refractory patients, namely, 15% CRR and time to progression of 6.8 months [38].

While theranostic approaches continue to evolve, including the use of various molecular targets beyond CD37, such as integrin receptors, HER2, claudin 18, and glutathione-sensing systems [39], the present study focuses on the clinical efficacy and safety of ^177^Lu–lilotomab satetraxetan in relapsed/refractory follicular lymphoma. Pretreatment imaging or dosimetry scans, such as those using ^111^In-labeled monoclonal antibodies, were not included for Betalutin, consistent with prior experience in radioimmunotherapy [40], where omitting pre-scans did not affect safety outcomes. This approach was chosen to reduce cost, simplify logistics, and make the treatment more readily available to patients, while still allowing for meaningful assessment of clinical efficacy and tolerability.

Since the PARADIGME study, novel treatments, such as CAR-T cell therapies, bispecific antibodies (BsAbs), antibody–drug conjugates (ADCs), and small-molecule inhibitors, have gained a significant market position. Some options, particularly PI3K and EZH2 inhibitors, have shown efficacy but raised concerns about immune-related toxicity. Among the most transformative advances are CAR-T therapies, initially developed for aggressive lymphomas but now also effective in indolent FL. In the ZUMA-5 trial, axi-cel achieved an ORR of 94% and a CRR of 79% with a median PFS of ~40 months [41]. Similarly, tisa-cel demonstrated an ORR of 86% and a CRR of 68% in the ELARA trial [42]. BsAbs have emerged as practical “off-the-shelf” options that redirect T cells to B cells. Mosunetuzumab, the first BsAb approved in FL, showed an ORR of 80% and a CRR of 60% in a Phase II trial [43]. Extended follow-up revealed durable responses beyond 3 years [44], with some partial responses deepening to CR [45,46]. Other BsAbs (glofitamab, epcoritamab, and odronextamab) have demonstrated similar activity [45,46,47,48,49,50,51]. Loncastuximab tesirine, an anti-CD19 ADC, showed an ORR of 97% and a CRR of 77% when combined with rituximab in a Phase II trial in relapsed FL, with rapid and deep responses [52]. Compared with historical standards, these novel agents provide high and durable CRRs and new hope for relapsed FL patients. Used earlier or in rational combinations, they may prolong disease control [53].

However, despite their promise, these emerging therapies come with specific challenges. CAR-T treatment is resource-intensive, requires specialized centers, and carries risks such as Cytokine Release Syndrome (CRS) and neurotoxicity, although these are less common in FL [54]. BsAbs, while more accessible, still pose risks for CRS and require prolonged administration, raising concerns about compliance and immunosuppression. Long-term B-cell depletion from CAR-T or continuous BsAb therapy may lead to infections or hypogammaglobulinemia, requiring supportive care. In this rapidly evolving treatment landscape, an important unmet need remains for effective therapies that can be delivered in less specialized settings and to patients who are elderly, frail, or refractory to multiple prior biologic therapies.

Due to premature termination prior to completion of the planned enrollment and follow-up, the study has certain limitations that should be considered when interpreting the results. Compared to the original study design, the reduced sample size and shorter observation period resulted in fewer events than would have been anticipated had the trial been completed as planned. For the ORR, the premature termination may have caused a preferential capture of early responders and may have therefore influenced the magnitude of the observed response estimates. The high proportion of informed censoring (non-random) further limits the robustness of time-to-event analyses. For antibody–radionuclide conjugate drugs, it is of particular interest to study the potential for long-term or delayed toxicities. This aspect could not be fully addressed due to the early termination. Furthermore, as a consequence of the early termination, the originally planned statistical analyses could not be fully implemented, and an abbreviated analysis approach was applied, with results largely based on observed data. Nevertheless, measures were taken to mitigate the impact of missing data for the primary endpoint, i.e., by ensuring that all patients enrolled were followed until the 12-week assessment. While these limitations warrant cautious interpretation, the results provide meaningful clinical insights and contribute valuable evidence to the understanding of safety and efficacy in this patient population.

In addition, the premature termination of the study also affected the interpretation of data from Part C. Part C was originally intended to enroll a larger cohort of patients with relapsed indolent NHL to further characterize pharmacokinetics and safety across related histologies. Due to early termination, only a limited number of patients were enrolled, precluding separate or statistically meaningful analyses of this cohort. Consequently, pharmacokinetic analyses were performed by pooling patients from Parts B and C, consistent with the approach used in Part A [29] and based on the shared CD37-targeting mechanism of Betalutin. For safety assessments, patients from Part C were included to capture relevant exposure-related events; however, efficacy results focus on the intended follicular lymphoma population of Part B, which provides the most representative and clinically relevant dataset for Betalutin.

Betalutin offers a distinct mechanism by targeting CD37 separately from CD19 and CD20. This allows potential activity even after the failure of other antigen-directed therapies, including CAR-T cells, bispecific antibodies, and anti-CD19-based ADCs. Preclinical data support its efficacy in resistant B-cell lymphoma lines, and current studies confirm its favorable safety profile. Preliminary clinical data combining Betalutin with rituximab also showed encouraging results, with 7/7 patients responding and 5/7 achieving CR [55]. Given its single-dose administration and manageable toxicity profile, Betalutin may be particularly suited for older or frail patients, those unable to undergo intensive treatments, and those treated in centers without access to CAR-T or prolonged BsAb administration.

## 4. Materials and Methods

### 4.1. Patients

The participants in the PARADIGME study were male or female, aged ≥ 18 years, with relapsed FL of grades I–IIIa. All patients were required to have less than 25% bone marrow infiltration of tumor cells, a baseline platelet count ≥ 150 ×10^9^/L, a life expectancy of at least 3 months, a World Health Organization (WHO) performance status ≤ 2, and disease measurable by radiological methods, and to follow contraceptive and pregnancy testing requirements. The patients were further required to have received at least two prior lines of therapy, including an anti-CD20 monoclonal antibody and an alkylating agent, and to be refractory to at least one previous regimen that contained rituximab or another anti-CD20 antibody. Following an interim analysis (see statistical analysis, below), an additional cohort (“special population”) allowed the enrollment of patients with (i) prior autologous SCT performed at least 2 years before enrollment and/or (ii) baseline platelet counts of 100–150 × 10^9^/L. The participants in Part C met eligibility criteria largely consistent with those of Part B. However, Part C expanded the eligible disease subtypes to include marginal zone lymphoma, small lymphocytic lymphoma, lymphoplasmacytic lymphoma, and mantle cell lymphoma.

### 4.2. Interventions

Betalutin (developed by Nordic Nanovector AS, Oslo, Norway) consisted of the anti-CD37 murine monoclonal antibody lilotomab conjugated to the chelator satetraxetan (p-SCN-benzyl-DOTA) that stably binds the β-emitting isotope 177Lu. The product was supplied in single-dose, ready-to-use vials. The specific activity of Betalutin was 100–500 MBq per mg at the time of injection, allowing an extended shelf life compatible with international shipment and logistics. Dosing, corrected for the physical decay of ^177^Lu, was provided according to body weight and in two different regimens, with cold lilotomab pre-dosing four hours prior to Betalutin: 40 mg of lilotomab and 15 MBq/kg of Betalutin (“40/15 regimen”) or 100 mg/m^2^ of lilotomab and 20 MBq/kg of Betalutin (“100/20 regimen”). Thus, one pretreatment regimen was based on a fixed dose of lilotomab, 40 mg, generating a more defined “combined specific activity”, i.e., the number of radioactive vs. non-radioactive molecules when combined with Betalutin. The other pretreatment regimen was instead related to the body surface of the patients and could therefore vary in the “combined specific activity” with the Betalutin.

**Figure 5:**
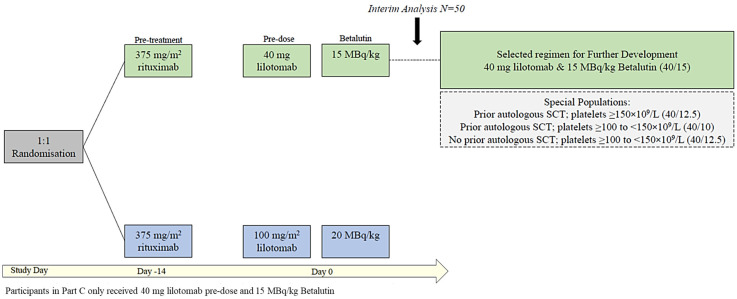
Study design LYMRIT-37-01 Part B (PARADIGME) and Part C.

As depicted in Figure 5, all the patients received pretreatment with 375 mg/m^2^ rituximab 14 days prior to the Betalutin administration (Day −14) to clear circulating peripheral B-lymphocytes from the blood and spleen and optimize the biodistribution of Betalutin. In the previously published Phase I study (Part A), different pretreatment time schedules with rituximab were tested [29]. The rituximab pretreatment was amended to include only one dose two weeks before Betalutin in the current study to simplify procedures and because the effect of repeated treatment was considered limited in the rituximab-refractory patient population included in the study.

### 4.3. Study Design and Endpoints

The patients were screened, and baseline images were obtained in the 4 weeks before rituximab pretreatment. On Day 1, eligible patients were randomized 1:1 to receive either the “40/15 regimen” or the “100/20 regimen”. These were compared because Part A data indicated that both regimens were relatively effective. Tumor responses were assessed at 3 and 6 months after treatment by contrast-enhanced computed tomography (CT), magnetic resonance imaging (MRI), or fluorodeoxyglucose (FDG)-positron emission tomography with CT (PET-CT) and thereafter by CT or MRI scans every 3 months for one year and then every 6 months until disease progression or the start of any further anti-cancer treatment. If bone marrow infiltration was present at baseline, repeat bone marrow biopsies were performed at months 3 and 6. Radiological responses were assessed centrally by an Independent Review Committee (IRC) according to [56], with the primary endpoint being ORR, defined as the proportion of patients achieving a best response of either complete response (CR) or partial response (PR) at any time. Secondary endpoints included complete response rate (CRR), duration of response (DoR), duration of complete response (DoCR), and PFS. DoR was defined as the time from first response (CR or PR) to documented disease progression, death, or study withdrawal. Patients who had not relapsed or progressed were censored at the last adequate tumor assessment.

Adverse events (AEs) and serious adverse events (SAEs) were collected via electronic case report forms from the signing of informed consent until the end of the treatment period, defined as 12 weeks after the administration of the study drug. Thereafter, only new onset AEs of special interest (AESIs) and treatment-related AEs and SAEs with onset >12 weeks after the administration of Betalutin were reported during the long-term follow-up period. The severity of AEs was graded according to the NCI-CTCAE version 4.0 [57]. As an antibody–radionuclide conjugate, Betalutin has a long-term risk of inducing secondary malignancies, so AESIs were defined as secondary cancers, including acute myelogenous leukemia, myelodysplastic syndrome, and aplastic anemia. Standard biochemistry and hematology clinical safety laboratory evaluations were performed periodically throughout the study. Weekly blood samples were required in all participants after Betalutin administration until platelet and neutrophil counts had recovered to ≥100 × 10^9^/L and ≥1.5 × 10^9^/L after the nadir, respectively.

To demonstrate the extent of CD37 expression in tumor tissue, immunohistochemistry staining was performed using the anti-human CD37 monoclonal antibody clone CT1 (Leica Biosystems Newcastle Ltd., Newcastle-upon-Tyne, UK). CD37 expression in tumor tissue was determined using an available archived tumor biopsy, or the participants were required to provide a new tumor tissue biopsy pre-dose.

Pharmacokinetic assessments, mandatory for patients in Part C and optional in Part B, were assessed by measuring the emission of low-energy gamma photons from the ^177^Lu radionuclide using a commercially available gamma counter (Wizard 3470, Perkin Elmer, Shelton, CT, USA). The radioactivity content was measured in blood samples taken at baseline (before lilotomab infusion) and up to week 5 (Figure 4).

### 4.4. Statistical Analyses and Adapted Design

A sample size of 65 patients in each of the two study arms, making a total of 130 patients, was planned based on results obtained in the subgroup of FL patients in Study Part A, with expected ORRs of 69% for the 40/15 arm and 42% for the 100/20 arm, respectively.

Following the first planned interim analysis of the ORR performed after 47 patients, the Safety Review Committee (SRC) recommended that only the 40/15 regimen be selected for further clinical development. Randomization was stopped, and further participants were enrolled into the 40/15 regimen only. Patients with a previous autologous or allogeneic SCT were originally excluded. However, as this interim analysis showed a mild toxicity profile, the eligibility criteria were widened to allow the enrollment of patients with (i) prior autologous SCT performed at least 2 years prior to enrollment and/or (ii) baseline platelet counts of 100–150 × 10^9^/L. For this “special population” in Part B, patients with prior SCT and platelets ≥ 150 × 10^9^/L and those without SCT but with platelets 100–150 × 10^9^/L received a 40 mg lilotomab + 12.5 MBq/kg Betalutin regimen, whereas those with prior SCT and platelets 100–150 × 10^9^/L received a 40 mg lilotomab + 10 MBq/kg Betalutin regimen (Figure 4). These participants were monitored carefully for at least 6 weeks to evaluate any dose-limiting toxicity (DLT).

As recruitment was slower than expected and impacted by the COVID-19 pandemic, a second interim efficacy analysis was conducted after 12 weeks of efficacy data were available from 61 patients in the 40/15 arm. With response rates lower than anticipated and no opportunity to meet the primary endpoint, even if all planned patients were recruited, the sponsor decided to prematurely terminate the study after all the patients who had been included at the time had reached the 12-week efficacy evaluation point. An abbreviated analysis was then conducted, with the efficacy analysis set consisting of the 100 patients from PARADIGME (Part B) who had received Betalutin and were assigned to either the 40/15 or the 100/20 regimen. The safety analysis set consisted of all the patients (*n* = 113) from Part B or Part C who received at least one dose of any study intervention (rituximab, lilotomab, or Betalutin). Figure 1 shows the detailed disposition of patients. Response data were determined based on the assessment by the IRC. Due to the premature termination, analyses were conducted in a descriptive manner only, with response rates and confidence intervals, but no hypothesis testing.

### 4.5. Ethical Aspects

The study protocol (EUDRACT No 2011-000033-36, approved on 18 October 2012) was approved by all the relevant regulatory agencies, independent ethics committees, or institutional review boards before the study began in each country and at each site (details see Supplementary Materials). All the patients gave their written informed consent before enrolment at each participating site. The study was conducted in accordance with the Declaration of Helsinki, International Conference for Harmonisation (ICH) Good Clinical Practice Guidelines (GCP), including ICH E6(R2), 2016 [58], and other applicable laws and regulations.

## 5. Conclusions

Betalutin demonstrates meaningful anti-tumor activity and a favorable safety profile in relapsed/refractory FL. While its efficacy is more evident earlier in the treatment course, its tolerability makes it a viable option for patients who are ineligible for more toxic therapies. Betalutin may have a role in future combined regimens with emerging immunotherapies, such as BsAbs, anti-CD19 agents, and immunomodulators.

## Figures and Tables

**Table 1:** Demographics and baseline characteristics for PARADIGME (Part B: efficacy analysis set).

Demographic/Variable	Statistic	40/15 *N* = 72	100/20 *N* = 28	Overall *N* = 100
Age	Mean (SD)	67.2 (9.9)	65.8 (9.2)	66.8 (9.7)
Age category	≥65 years; *n* (%)	46 (64)	19 (68)	65 (65)
Gender *n* (%)	Females; *n* (%)	43 (60)	16 (57)	59 (59)
	Males; *n* (%)	29 (40)	12 (43)	41 (41)
Weight (kg)	Mean (SD)	75.3 (15.9)	73.0 (16.7)	75.7 (16.1)
Time since initial diagnosis (months)	Mean (SD)	92.7 (73.29)	85.2 (59.4)	90.6 (69.4)
FL grade; *n* (%)	Grade I	13 (18.1%)	6 (21.4%)	19 (19.0%)
	Grade II	31 (53.1%)	11 (39.3%)	42 (42.0%)
	Grade IIIA	23 (31.9%)	9 (32.1%)	32 (32.0%)
Time since most recent relapse (months)	Mean (SD)	2.6 (4.0)	1.7 (1.7)	2.3 (3.4)
Number of prior systemic therapies	Mean (SD)	3.1 (1.6)	3.1 (1.2)	3.1 (1.5)
	Median (range)	3 (1, 12)	3 (2, 6)	3 (1, 12)
≥2 prior systemic therapies	*N* (%)	71 (99)	28 (100)	99 (99)
Time from last previous treatment to study intervention (months)	Mean (SD)	9.9 (14.1)	4.7 (4.8)	8.4 (12.5)
	Median (range)	4.9 (1.1, 95.9)	3.2 (1.1, 22.1)	4.4 (1.1, 95.9)
Last therapy refractory	*n*, %	52 (72)	24 (86)	76 (76)
Refractory to rituximab/anti-CD20	*n*, %	70 (97.2%)	25 (90)	95 (95)
Refractory to ≥2 therapies for FL	*n*, %	36 (50)	19 (68)	55 (55)

Abbreviations: FL = follicular lymphoma; SD = standard deviation.

**Table 2:** Best overall response (Part B: efficacy analysis set).

Best Overall Response Assessed by IRC	40/15 *N* = 72	100/20 *N* = 28
CR, *n* (%)	15 (20.9%)	4 (14.3%)
PR, *n* (%)	13 (18.1%)	5 (17.9%)
SD, *n* (%)	21 (29.2%)	11 (39.3%)
PD, *n* (%)	21 (29.2%)	7 (25.0%)
Not evaluable or not performed, *n* (%)	2 (2.8%)	1 (3.6%)
ORR, *n* (%), [95% CI]	28 (38.9%) [27.6%, 51.1%]	9 (32.1%) [15.9%, 52.4%]
CRR, *n* (%), [95% CI]	15 (20.8%) [12.2%, 32.0%]	4 (14.3%) [4.0%, 32.7%]

Abbreviations: complete response = CR; partial response = PR; progressive disease = PD; ORR = overall response rate; CRR = complete response rate.

**Table 3:** Duration of response and duration of complete response (Part B: efficacy analysis set).

Statistic	Response Based on CR or PR		Response Based on CR	
	40/15 *N* = 72	100/20 *N* = 28	40/15 *N* = 72	100/20 *N* = 28
Participants included in the analysis	28	9	15	4
Participants with events	12	8	6	3
Participants censored	16	1	9	1
DoR 3 months (%)	86.4%	88.9%	100%	100%
DoR 6 months (%)	60.2%	33.3%	77.8%	50.0%
DoR 9 months (%)	43.9%	33.3%	46.7%	50.0%
DoR 12 months (%)	43.9%	33.3%	46.7%	50.0%
DoR 15 months (%)	43.9%	22.2%	46.7%	25.0%
Median DoR (months)	8.0	3.4	8.5	9.2

Abbreviations: CR = complete remission; PR = partial remission; DoR = duration of response.

**Table 4:** Treatment-emergent adverse events (occurring in >3.0% of patients; Parts B and C: safety analysis set).

Preferred Term TEAE	Number (%) of Patients			
	40/12.5 *N* = 9	40/15 *N* = 76	100/20 *N* = 28	Overall *N* = 109
Thrombocytopenia	3 (33.3%)	13 (17.1%)	3 (10.7%)	19 (16.8%)
Anemia	2 (22.2%)	11 (14.5%)	5 (17.9%)	18 (15.9%)
Neutropenia	3 (33.3%)	12 (15.8%)	3 (10.7%)	18 (15.9%)
Fatigue	2 (22.2%)	8 (10.5%)	3 (10.7%)	13 (11.5%)
Nausea	0	9 (11.8%)	2 (7.1%)	11 (9.7%)
Diarrhea	0	7 (9.2%)	2 (7.1%)	9 (8.0%)
Abdominal pain	0	5 (6.6%)	2 (7.1%)	7 (6.2%)
Arthralgia	1 (11.1%)	2 (2.6%)	4 (14.3%)	7 (6.2%)
Back pain	0	5 (6.6%)	2 (7.1%)	7 (6.2%)
COVID-19	1 (11.1%)	5 (6.6%)	0	6 (5.3%)
Lymphopenia	1 (11.1%)	4 (5.3%)	1 (3.6%)	6 (5.3%)

Abbreviations: TEAE = treatment-emergent adverse event.

## Data Availability

The data presented in this study are available on request from the corresponding author. (Data are not publicly available due to privacy restrictions).

## Supplementary Materials

The following supporting information can be downloaded at: https://www.mdpi.com/article/10.3390/ph19020250/s1, List of Independent Ethics Committees and/or Institutional Review Boards.

## Abbreviations

The following abbreviations are used in this manuscript:

^131^I	Iodine-131 (radioisotope used in RIT)
^177^Lu	Lutetium-177 (radioactive isotope used in radiotherapy)
^90^Y	Yttrium-90 (radioisotope used in RIT)
ADC	Antibody–Drug Conjugate
AE	Adverse Event
AESI	Adverse Event of Special Interest
axi-cel	Axicabtagene Ciloleucel (CAR-T therapy)
BSA	Body Surface Area
BsAb	Bispecific Antibody
CAR-T	Chimeric Antigen Receptor T-cell therapy
CD19	Cluster of Differentiation 19 (B-cell surface antigen)
CD20	Cluster of Differentiation 20 (B-cell surface antigen)
CD3	Cluster of Differentiation 3 (T-cell surface antigen)
CD37	Cluster of Differentiation 37 (B-cell surface antigen)
CHOP	Chemotherapy regimen: Cyclophosphamide, Doxorubicin, Vincristine, Prednisone
CI	Confidence Interval
COVID-19	Coronavirus Disease
CR	Complete Response
CRR	Complete Response Rate
CRS	Cytokine Release Syndrome
CT	Computed Tomography
CTCAE	Common Terminology Criteria for Adverse Events
DOTA	1,4,7,10-tetraazacyclododecane-1,4,7,10-tetraacetic acid (chelator)
DLT	Dose-Limiting Toxicity
DNA	Deoxyribonucleic Acid
DoCR	Duration of Complete Response
DoR	Duration of Response
EUDRACT	European Union Drug Regulating Authorities Clinical Trials Database
EZH2	Enhancer of Zeste Homolog 2 (target of small-molecule inhibitors)
FDA	U.S. Food and Drug Administration
FDG-PET	Fluorodeoxyglucose Positron Emission Tomography
FL	Follicular Lymphoma
GCP	Good Clinical Practice
ICH	International Council for Harmonisation of Technical Requirements for Pharmaceuticals for Human Use
ICH E6(R2)	ICH Guideline for Good Clinical Practice, Revision 2
ICANS	Immune Effector Cell-Associated Neurotoxicity Syndrome
IRC	Independent Review Committee
liso-cel	Lisocabtagene Maraleucel (CAR-T therapy)
MBq/kg	Megabecquerel per kilogram (radioactivity dosage per body weight)
MeV	Mega Electron Volt (unit of energy)
MRI	Magnetic Resonance Imaging
NCI-SEER	National Cancer Institute—Surveillance, Epidemiology, and End Results
NHL	Non-Hodgkin Lymphoma
ORR	Overall Response Rate
PFS	Progression-Free Survival
PI3K	Phosphoinositide 3-Kinase
PD	Progressive Disease
POD24	Progression of Disease within 24 months
PR	Partial Response
RIC	Radioimmunoconjugate
RIT	Radioimmunotherapy
r/r	Relapsed or Refractory
SAE	Serious Adverse Event
SCN	Isothiocyanate (as in p-SCN-benzyl-DOTA chelator)
SCT	Stem Cell Transplant
SD	Stable Disease
SRC	Safety Review Committee
TEAE	Treatment-Emergent Adverse Event
tisa-cel	Tisagenlecleucel (CAR-T therapy)
WHO	World Health Organization
