# Supplementary material for: A Phase II Study of 177Lu–Lilotomab Satetraxetan, a CD37 Antibody–Radionuclide Conjugate, as Third- or Later-Line Treatment of Rituximab-Refractory Follicular B-Cell Lymphoma Patients
Source: Pharmaceuticals (Basel). 2026 Feb 1;19(2):250. doi: 10.3390/ph19020250 (PMC12943636; doi:10.3390/ph19020250)
Supplement: Supplementary file 1 [file pharmaceuticals-19-00250-s001.zip › pharmaceuticals-4048733-supplementary.pdf]

List of Independent Ethics Committees and/or Institutional Review Boards

The IECs/IRBs in the following table gave a favorable opinion for the study for sites that were subsequently initiated and screened participants in the LYMRIT-37 Part B and C studies.

IEC/IRB Details	Approval Date	Approval Code
REK Regional Committees for Medical and Professional Health Research Ethics, Gullhaugveien 1-3, 0484 Oslo, Norway	29.May.2018	2018/731/REK
The Regional Ethical Review Board in Umeå Department of Medical Research Samverkanshuset Universitetsområdet 901 87 Umeå, Sweden	22.Feb.2018	2018-38-32M
REK Regional Committees for Medical and Professional Health Research Ethics, Gullhaugveien 1-3, 0484 Oslo, Norway	29.May.2018	2018/731/REK
Office Committees Northern Ireland (ORECNI), Business Services Organisation Lissue Industrial Estate West, Moira Road, Lisburn, BT28 3RF. Northern Ireland (UK)	26. Feb.2016	14/NI/1037
Ethics Committee of Olomouc University Hospital and the Faculty of Medicine of Palacký University, Olomouc I. P. Pavlova 6, 775 20 Olomouc, Czech Republic	18.Sep.2017	103/14 MEK 12
Ethics Committee of Olomouc University Hospital and the Faculty of Medicine of Palacký University, Olomouc I. P. Pavlova 6, 775 20 Olomouc, Czech Republic.	18.Sep.2017	103/14 MEK 12
Office Committees Northern Ireland (ORECNI), Business Services Organisation Lissue Industrial Estate West, Moira Road, Lisburn, BT28 3RF. Northern Ireland (UK)	26. Feb.2016	14/NI/1037
Office Committees Northern Ireland (ORECNI), Business Services Organisation Lissue Industrial Estate West, Moira Road, Lisburn, BT28 3RF. Northern Ireland (UK)	26.Feb.2016	14/NI/1037

Ethics Committee of the State of<br>Upper Austria,<br>Wagner-Jauregg-Weg 15, 4021 Linz, Austria	18. Oct. 2021	A-108-14
Komisja Bioetyczna,<br>przy Okregowej Isbie Lekarskiej w Krakowie, ul.<br>Krupnicza 11a, 31-123 Krakow, Poland.	10. Feb. 2016	104/KBL/OIL/2015
Office Committees Northern Ireland<br>(ORECNI), Business Services<br>Organisation Lissue Industrial Estate West, Moira Road,<br>Lisburn, BT28 3RF.<br>Northern Ireland (UK)	26. Feb.2016	14/NI/1037
Komisja Bioetyczna,<br>przy Okregowej Isbie Lekarskiej w Krakowie, ul.<br>Krupnicza 11a, 31-123 Krakow, Poland.	24 February 2016	104/KBL/OIL/2015
Ethics Committee for Drug Research of Puerta de Hierro Majadahonda<br>University Hospital,<br>Northwest Area,<br>C/Joaquin Rodrigo, 2 28222 Majadahonda,<br>Madrid, Spain.	12. Sep.2016	EC-01 CA-01
Office of Research services<br>University of Tasmania<br>Private Bag 1<br>Hobart Tasmania 7001<br>Australia	24.Jan.2019	H0017076
National Institute of Pharmacy and<br>Nutrition<br>Division of Clinical Trials<br>1051 Budapest,<br>1372 Postafiók 450<br>Hungary	25. July.2018	OGUEI/32773-5/2018
Comitato Etico di Area Vasta Emilia<br>Centro<br>Via Albertoni, 15 40138 BOLOGNA<br>(BO)<br>Italy	07. Mar.2019	EM130-<br>2019_72/2014/U/Spe<br>r/AOUBo

Bâtiment ex USNB (RDC)<br>6 rue du Professeur Laguesse<br>CHRU LILLE [Lille University Hospital] CS70001<br>59037 Lille CEDEX<br>France	28.June 2018	EC 17/90
Bâtiment ex USNB (RDC)<br>6 rue du Professeur Laguesse<br>CHRU LILLE [Lille University Hospital] CS70001<br>59037 Lille CEDEX<br>France	15.Feb.2018	EC 17/90
Regionssekretariatet Juridisk kontor<br>De Videnskabsetiske Komitéer For Region Midtjylland Skottenborg 26<br>DK-8800 Viborg<br>Denmark	30.May.2018	EudraCT nr.: 2011-000033-36
Ethics Committee for Clinical Pharmacology (Eccp)<br>1051 Budapest, Zrínyi utca 3.<br>Levélcím: 1372 Postafiók 450<br>Hungary	25. July.2018	OGYÉI/32773-5/2018
Comité De Ética De La Investigación Con Medicamentos Del Hospital Universitario Puerta De Hierro Majadahonda<br>Spain	12. Sep.2016	EC-01 CA-01<br>Code: Lymrit-37-01<br>2011-000033-36
Rambam Health Care Campus<br>Helsinki Committee<br>Rambam Health Care Campus Haifa,<br>Israel	23. Jun.2019	0009-18-RMB
Comitato Etico dell'Area Vasta Emilia Nord<br>Viale Umberto I, 50 - Reggio Emilia Italy	22. May.2018	185/2018/FARM/IRCCSRE
Clinical Research Ethics Committee<br>Lancaster Hall<br>6 Little Hanover Street<br>Cork<br>Ireland	10. Apr.2020	Ref ECM 5 (2) 22/01/18 & ECM 3 (jjjjj) 09/04/2020
Ankara University Faculty of Medicine<br>Clinical Studies Ethics Committee<br>Morphology Building, 06100 Sıhhiye/ Ankara<br>Turkey	22. Jun. 2018	02-79-18

Comité De Ética De La Investigación<br>Con Medicamentos Del Hospital<br>Universitario Puerta De Hierro<br>Majadahonda<br>Spain	12. Sep.2016	EC-01 CA-01<br>Code: Lymrit-37-01<br>2011-000033-36

Comité de Ética de la Investigación<br>Con Medicamentos Del Hospital<br>Universitario Puerta De Hierro<br>Majadahonda<br>Spain	12. Sep.2016	EC-01 CA-01<br>Code: Lymrit-37-01<br>2011-000033-36
Helsinki Committee<br>Bnai Zion Medical Center<br>Helsinki<br>Finland	29. Aug.2018	0007-18-BNZ
Clinical Research Ethics Committee<br>Lancaster Hall<br>6 Little Hanover Street<br>Cork<br>Ireland	10. April.2020	Ref ECM 5 (2)<br>22/01/18 & ECM 3<br>(jjjjj) 09/04/2020
Clinical Research Ethics Committee<br>Lancaster Hall<br>6 Little Hanover Street<br>Cork<br>Ireland	10.April.2020	Ref ECM 5 (2)<br>22/01/18 & ECM 3<br>(jjjjj) 09/04/2020
Comité De Ética De La Investigación<br>Con Medicamentos Del Hospital<br>Universitario Puerta De Hierro<br>MajadahondA<br>Spain	12. Sep.2016	EC-01 CA-01<br>Code: Lymrit-37-01<br>2011-000033-36
Comité De Ética De La Investigación<br>Con Medicamentos Del Hospital<br>Universitario Puerta De Hierro<br>Majadahonda<br>Spain	12. Sep.2016	EC-01 CA-01<br>Code: Lymrit-37-01<br>2011-000033-36
Comitato Etico degli IRCCS Istituto<br>Europeo di Oncologia e Centro<br>Cardiologico Monzino<br>Via Ripamonti 435, 20141 Milan<br>Italy	01.Jun.2018	IEO 826 – RE1548/NC
Medical Ethics Committee Ghent University Hospital<br>C.<br>Heymanslaan 10 | B 9000 Ghent<br>Belgium	13. Jun.2018	2018/0604

Comitato Etico Irst Irccs Avr<br>Via Piero Maroncelli 40, 47014<br>Meldola (FC)<br>Italy	16. May.2018	3752/2018
Bâtiment ex USNB (RDC)<br>6 rue du Professeur Laguesse<br>CHRU LILLE [Lille University<br>Hospital] CS70001<br>59037 Lille CEDEX<br>France	15 Feb 2018	EC 17/90
Regionssekretariatet Juridisk kontor<br>De Videnskabsetiske Komitéer For<br>Region Midtjylland Skottenborg 26<br>DK-8800 Viborg<br>Denmark	24.Nov.2021	EudraCT no.: 2011-000033-36<br><br>Case no. 1-10-72-44-18

Medical Ethics Committee Ghent University Hospital<br>C.<br>Heymanslaan 10 | B 9000 Ghent<br>Belgium	13.June.2018	2018/0604
University Health Network Research<br>Ethics Board 10th Floor, Room 1056<br>700 University Ave. Toronto, Ontario,<br>M5G 1Z5<br>Canada	14. Sep.2018	18-5245
WCG IRB<br>1019 39th Avenue SE,<br>Suite 120 Puyallup, WA 98374<br>USA	23. Dec.2019	IRB Tracking<br>Number: 20180280<br>IRB registration<br>number:<br>IRB00000533
Medical Ethics Committee Ghent University Hospital<br>C.<br>Heymanslaan 10 | B 9000 Ghent<br>Belgium	13.June.2018	2018/0604
WCG IRB<br>1019 39th Avenue SE,<br>Suite 120 Puyallup, WA 98374<br>USA	23. Dec.2019	IRB Tracking<br>Number: 20180280<br>IRB registration<br>number:<br>IRB00000533
HUS:n eettiset toimikunnat Eettinen toimikunta III<br>Biomedicum Helsinki 2<br>C, 7. kerros Tukholmankatu 8 C 00290 Helsinki	06.Mar.2019	HUS/950/2018
HUS:n eettiset toimikunnat Eettinen toimikunta III<br>Biomedicum Helsinki 2<br>C, 7. kerros Tukholmankatu 8 C 00290 Helsinki	06.Mar.2019	HUS/950/2018

Bâtiment ex USNB (RDC)<br>6 rue du Professeur Laguesse<br>CHRU LILLE [Lille University<br>Hospital] CS70001<br>59037 Lille CEDEX<br>France	15.Feb.2018	EC 17/90
Medisch Ethische Toetsingscommissie (METC) UMCG Attn. Prof. dr. W.A. Kamps<br>Building De Brug, room 7.067 {LA15,<br>ih floor) Hanzeplein 1<br>9713 GZ, Groningen<br>The Netherlands	23.July.2018	METc 2018/179
WCG IRB<br>1019 39th Avenue SE,<br>Suite 120 Puyallup, WA 98374	23. Dec.2019	IRB Tracking Number: 20180280<br>IRB registration number:<br>IRB00000533
WCG IRB<br>1019 39th Avenue SE,<br>Suite 120 Puyallup, WA 98374	23. Dec.2019	IRB Tracking Number: 20180280<br>IRB registration number:<br>IRB00000533
Ankara University Faculty of Medicine<br>Clinical Studies Ethics Committee<br>Ankara University Faculty of Medicine<br>Morphology Building, 06100 Sıhhiye/ Ankar	22.Jan.2018	Decision No. 02-79-18

Office for Research Ethics Committees<br>Northern Ireland (ORECNI) Customer<br>Care & Performance Directorate<br>Lissue Industrial Estate West<br>Rathdown Walk Moira Road Lisburn<br>BT28 2RF<br>Northern Ireland (UK)	26.Feb.2016	14/NI/1037
University of Pittsburgh<br>Human Research Protection Office<br>(IRB)<br>3500 Fifth Avenue, Suite 106<br>Pittsburgh, PA 15213	14.June. 2019	STUDY19020374
WCG IRB<br>1019 39th Avenue SE,<br>Suite 120 Puyallup, WA 98374	23. Dec.2019	IRB Tracking Number: 20180280<br>IRB registration number:<br>IRB00000533

Ethics Committee of the University of Würzburg<br>Institute of Pharmacology and Toxicology Versbacher Str. 9 97078 Würzburg	23.Sep.2020	184/19_ff-am
Western University Health Sciences Research Ethics Board (HSREB)<br>Room 5150 Support Services Building 1393 Western Road<br>London, Ontario, Canada	17. June. 2021	Project ID: 113640 Reference Number/ID: LYMRIT-37-01 Amendment # 15 and IB 13.1
University of California San Francisco Medical Center<br>400 Pranassus Avenue<br>Box 0324<br>San Francisco, CA 94143<br>Please contact the UCSF IRB at 415476-1814 with any questions.<br>irb.ucsf.edu	27. Nov.2019	19-27495
Office for Research Ethics Committees Northern Ireland (ORECNI) Customer Care & Performance Directorate<br>Lissue Industrial Estate West<br>Rathdown Walk Moira Road Lisburn<br>BT28 2RF<br>Northern Ireland (UK)	26.Feb.2016	14/NI/1037
Office for Research Ethics Committees Northern Ireland (ORECNI) Customer Care & Performance Directorate<br>Lissue Industrial Estate West<br>Rathdown Walk Moira Road Lisburn<br>BT28 2RF<br>Northern Ireland (UK)	26. Feb.2016	14/NI/1037
Bâtiment ex USNB (RDC)<br>6 rue du Professeur Laguesse<br>CHRU LILLE [Lille University Hospital] CS70001<br>59037 Lille CEDEX<br>France	15. Feb.2018	EC 17/90
